# Beneficial Potential of *Banha-Sasim-Tang* for Stress-Sensitive Functional Dyspepsia via Modulation of Ghrelin: A Randomized Controlled Trial

**DOI:** 10.3389/fphar.2021.636752

**Published:** 2021-04-20

**Authors:** Sul-Ki Kim, Jin-Yong Joung, Yo-Chan Ahn, In-Chul Jung, Chang-Gue Son

**Affiliations:** ^1^ Liver and Immunology Research Center, College of Korean Medicine, Daejeon University, Daejeon, Republic of Korea; ^2^ Department of Health Service Management, Daejeon University, Daejeon, Republic of Korea; ^3^ Departments of Neuropsychiatry, College of Korean Medicine, Daejeon University, Daejeon, Republic of Korea

**Keywords:** functional dyspepsia, banha sasim-tang, herbal mecidine, ghrelin, clinical trial

## Abstract

**Background:** Functional dyspepsia (FD) is a highly complex pathophysiologic disease, which shows low recovery and high relapse rates. Therefore, a growing number of clinicians and patients are looking for alternative herbal medicine. *Banha-Sasim-Tang* (BST) is an herbal prescription for treating a wide range of dyspepsia in traditional Korean medicine (TKM).

**Aim of the study:** This study aimed to evaluate the efficacy of BST on FD in patients with psychological stress-related symptoms and to investigate the involvement of ghrelin. This study is registered at https://cris.nih.go.kr/with the identification number KCT 0002811.

**Materials and methods:** A total of 31 participants with FD who met the Rome IV criteria and the psychological stress-related pattern were enrolled in a double-blind, randomized, and controlled study. Participants were randomly assigned to the BST group (10 g twice daily for 4 weeks) or placebo group. The primary endpoint was a change in the Nepean Dyspepsia Index–Korean (NDI-K) score. The secondary endpoints were changes in the Visual Analog Scale (VAS) scores and plasma ghrelin level.

**Results:** All participants completed the study (*n* = 15 for BST, *n* = 16 for placebo). BST decreased NDI-K scores compared with placebo, but the difference was not statistically significant (37.40 ± 27.40 *vs* 22.50 ± 23.85, *p* = 0.12). VAS scores and plasma total ghrelin levels were significantly improved in patients who were treated with BST (3.19 ± 1.60 *vs* 1.38 ± 2.85, *p* = 0.03 for VAS and 105.69 ± 287.89 *vs* -142.31 ± 314.32, *p* = 0.03 for total ghrelin). No BST-related adverse effects were observed during the trial.

**Conclusion:** Our results indicate the clinical potential of BST for FD patients and are the first study to show the modulation of plasma ghrelin as one of its corresponding mechanisms.

**Clinical Trial Registration:** https://cris.nih.go.kr/, identifier KCT 0002811.

## Introduction

Functional dyspepsia is a recurrent or persistent dyspeptic symptom group that lasts more than 6 months but does not have explainable organic causes (Stanghellini et al., 2016). Several pathogeneses of FD have been reported, such as *Helicobacter pylori* or other infections, visceral hypersensitivity, low-grade mucosal inflammation, delayed gastric emptying (Talley and Walker, 2019). This highly complex pathology limits conventional therapy, and a growing number of clinicians and patients are alternatively looking for herbal medicine from a personalized and multitarget medical outlook (Chiarioni et al., 2018).

*Banha-Sasim-Tang*, also known as Banxia-Xiexin-Tang in traditional Chinese medicine and Hange-Shashin-To in Kampo medicine, is a traditional herbal medicine that has widely been used for treating gastrointestinal disease in traditional Korean medicine (Fan et al., 2019). Several clinical studies have shown the beneficial effects of BST against gastric dyspepsia and esophageal reflux symptoms (Gan et al., 2014; Shin et al., 2017). In particular, a preclinical study showed that BST-derived improvement in gastric emptying capacity might be linked to activated secretion of ghrelin, a peptide hormone known to promote appetite (Klok et al., 2007; Wu et al., 2014). Clinical studies presented the lowered plasma levels of ghrelin in patients with FD (Takakazu et al., 2013; Choi et al., 2017). Accordingly, we supposed that the underlying mechanism of BST on relieving FD symptoms comes by modulating ghrelin.

In practices of TKM/TCM, clinicians have adapted the pattern identification in processes of diagnosis and treatment for certain disorders including FD, which stresses the cluster of symptoms with its affecting factors (Ko et al., 2014). FD has generally divided into two subgroups as postprandial distress syndrome (PDS) and epigastric pain syndrome (EPS) (Houte et al., 2020), in conventional medicine, while TKM/TCM generally classify FD into six different identifications (Kim et al., 2010). Of those, the “liver Qi invading stomach (肝气犯胃)” pattern is most common in Korean FD patients (approximately 52.9%) (Park et al., 2008). This pattern is characterized by psychological stress-related factors that induce or accelerate the FD symptoms, including epigastric distension and pain extending to sides, belching, sighing, or nausea (Zhao et al., 2018, detail in Supplementary Tables S1, S2). In fact, stress is one of the key inducers of FD symptoms, and an anti-stress strategy is the main management approach for FD patients (Labanski et al., 2020).

On the other hand, the objective assessment of symptom severity frequently becomes an important issue especially for subjective disorders likely FD (McColl, 2004). Many instruments have been developed, but Nepean Dyspepsia Index has been mostly used as a validated questionnaire evaluating dyspepsia-specific symptoms and related quality of life (Talley et al., 1999). The Korean version of NDI has been validated (Lee et al., 2003) and properly adapted in various FD-related clinical trials in Korea (Yoon et al., 2018; Park et al., 2019). One clinical study reported the FD prevalence of approximately 25% of the general population, whose average of NDI-K score was 51.06 ± 29.64 (Ko et al., 2018).

In this study, we aimed to evaluate the efficacy and safety of BST in the FD patient having “liver Qi invading stomach (肝气犯胃)” pattern and to investigate its underlying mechanism related to ghrelin.

## Materials and Methods

### Participants

Participants who met the following key eligibility criteria were included: age between 19 and 65 with a diagnosis of FD, moderate or severe degree according to the Rome IV criteria and corresponding to “liver Qi invading stomach” pattern (Table 1). The pattern identification including “liver Qi invading stomach” was determined using a questionnaire which was developed in 2010 in Korea (Kim et al., 2010). Briefly, FD patient belongs to one of six patterns by the highest sum score weighted for 33 symptom-related questions and clinician-derived diagnosis. Details of pattern identification and weight scores are shown in Supplementary Tables S1, S2. Also, participants were included if he/she had at least two symptoms of 2 (moderate) or 3 (severe) score with total score ≥6 points using the short form of NDI-K. The short form of NDI-K was modified from the original NDI-K; using key 8 items of 15 and scoring 0 (no symptom) to 3 (severe), respectively (Supplementary Table S3). The cut-off score (total score ≥6) has been decided by our research team based on the previous study results, which reported the total score ≥6 is enough for the moderate level of FD complaining (Fraser et al., 2007). The key exclusion criteria were participants who had organic abnormalities in gastric endoscopy. Participants currently using or who had used nonsteroidal anti-inflammatory drugs (NSAIDs) or corticosteroids were excluded. Details of the inclusion and exclusion criteria are provided in Table 1. The protocol was approved by the institutional review board (IRB) at Daejeon Korean Medicine Hospital (IRB number: DJDSKH-17-DR-25–2). And this study is registered at https://cris.nih.go.kr/with the identification number KCT 0002811.

**Table 1:** Inclusion and exclusion criteria.

The inclusion criteria are as follows
1) participants who are 19–65 years old
2) participants who verified no evidence of structural disease through endoscopy within the last 24 months
3) participants who meet the functional dyspepsia according to rome Ⅳ criteria (criteria fulfilled for the last 3 months with symptom onset at least 6 months before diagnosis, more than one of the followings)
A. Bothersome postprandial fullness
B. Bothersome early satiation
C. Bothersome epigastric pain
D. Bothersome epigastric burning
4) participants who meet the “liver qi invading stomach” with Korean medicine pattern identification (see Supplementary Tables S1, S2)
5) participants who have at least two moderate symptoms or more than the total score of 6 among the 8 items of short form of NDI-K. (See Supplementary Table S3)
The exclusion criteria are as follows
1) participants with a history of gastrointestinal surgery (except for appendectomy)
2) participants at risk of gastrointestinal motility, including gastrointestinal bleeding, mechanical intestinal obstruction, or perforation, etc
3) participants with diseases that may affect evaluation of efficacy in the previous 2 years
For example, gastroduodenal ulceration, gastric cancer, or colorectal cancer, etc
4) participants who need anti-bactericidal treatment or are under anti-bactericidal treatment for *H. pylori*
5) participants who have taken medication affecting evaluation of trial medication efficacy within 1 month before the screening visit
For example, glucocorticosteroids, nonsteroidal anti-inflammatory drugs, or prokinetics, etc
6) participants whose AST or ALT is more than 2 times the upper limit of normal, or creatinine is more than 1.2 times the upper limit of normal
7) woman who are pregnant or lactating
8) participants who have participated in other clinical trials and taken clinical trial medication within the last 3 months

NDI-K: Nepean dyspepsia Index-Korean, AST: Aspartate aminotransferase, ALT: Alanine aminotransferase

### Trial Design and Intervention

**FIGURE 1:**
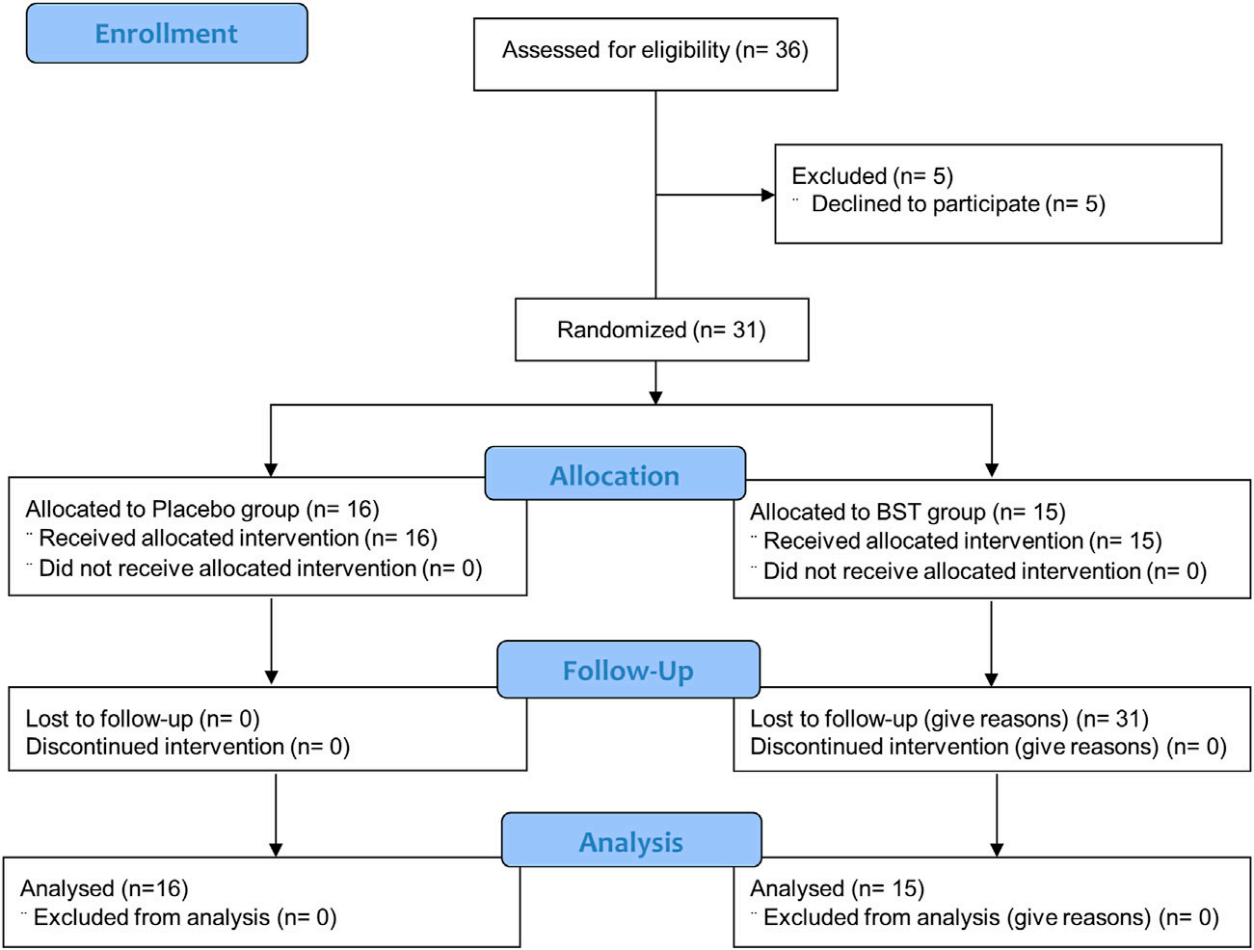
Study flow diagram.

This study was a double-blind, placebo-controlled, randomized, single-center trial. Participants were randomly assigned at a 1:1 ratio to receive 10 g of BST at once and orally took twice a day or matching placebo for 4 weeks, with a 2 weeks follow-up period without treatment (Figure 1). The random allocation number was handled by independent statistical experts and blocked random assignments were used. Every 2 weeks after taking BST or placebo, any unconsumed medications were returned to ensure medication adherence.

**FIGURE 2:**
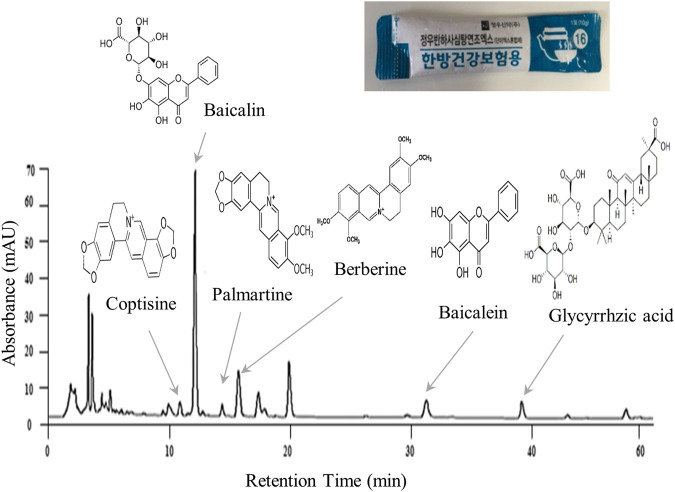
Molecular fingerprinting of *Banha-Sasim-Tang*.

BST and placebo were prepared in syrup form by Jeong-Woo Pharmaceutical Company Ltd. according to Korean Good Clinical Practice guideline. BST consists of 7 different types of TKM-derived herbs (Table 2). Briefly, seven herbal materials were extracted by boiling water, and extracts were filtered, concentrated, and lyophilized. Molecular fingerprinting of BST (Figure 2) was performed using a high-performance liquid chromatography system (HPLC, Agilent 1,100 system, CA, United States) equipped with an autosampler (G11313A), column oven (GA1316A), binary pump (G1312), diode-array-detector (DAD), and degasser (GA1379A) as described previously A matching placebo was prepared in a form, flavor, and fragrance very similar to BST and contained corn starch, caramel pigment, etc. (Table 2). The participants were provided the trial drugs labeled with the number of the trial protocol (DJOMCBST-201701), product name (BST or placebo), batch number (803), expiration date (2021.02.18), address, contact number of the principal investigator, and precaution “Keep out of reach of children.”

**TABLE 2:** Components of *Banha-Sasim-Tang* and Placebo in one syrup.

*Banha-Sasim-Tang*		Placebo	
Scientific name	Amount (g)	Scientific name	Amount (g)
*Pinellia ternate* (thunb.) makino	1.18	Corn starch	1.92
*Scutellaria baicalensis* georgi	0.84	Caramel pigment	0.08
*Panax ginseng* C.A. Meyer	0.80		
*Glycyrrhiza uralensis* Fisch	0.73		
*Zingiber officinale* roscoe	0.58		
*Coptis chinensis* franch	0.13		
*Ziziphus jujuba* mill	0.51		
Beta-cyclodextrin	1.50	Beta-cyclodextrin	1.50
Apple concentrate	1.35	Apple concentrate	1.35
Sodium carboxymethyl cellulose	0.05	Sodium carboxymethyl cellulose	0.05
Sodium benzoate	0.01	Sodium benzoate	0.01
Purified water	Suitable amount	Purified water	Suitable amount
Total	10.00		10.00

### Assessment

The primary endpoint was the change in the NDI-K score, and the changes in Visual Analogue Scale scores as a secondary endpoint. These measurements were assessed at time points of baseline, 4 weeks treatment and 2 weeks follow-up, respectively. In addition, we measured the plasma levels of acyl-ghrelin, des-acyl-ghrelin, and total ghrelin. At both time points of the baseline and of the one day after the last drug taken, venipunctures were conducted under an 8 h fasting state. The blood samples were used for liver and kidney function tests, and complete blood cell count test to ensure stability for medication as well as for measurement of ghrelin. One part of the blood sample was collected at 3.5 ml SST tube (BD Vacutainer®) and left at room temperature for 30 min for plasma ghrelin level analysis. Samples were centrifuged at 3,000 rpm for 20 min and dispensed 0.5 ml each for primary and back-up tubes. The tubes were stored at −80°C and sent to one external institution (SML Meditree Co., Ltd., Seoul, Korea) for determinations of plasma ghrelin levels using a human acyl-ghrelin ELISA kit (Abbexa®) and human des-acyl-ghrelin ELISA kit (MyBioSource.Inc.).

### Sample Size Estimation

This study aimed to assess the efficacy of BST in patients with FD. The formula for estimating the sample size for each arm is as follows:

$$\text{n}=\frac{2{\left(z_{\alpha 2}+z_{1-\beta }\right)}^{2}\delta_{d}^{2}}{{\left(\mu_{d}\right)}^{2}}.$$

The average change in the NDI-K symptom score was assumed to be 17.40 considering the appropriate sample size, according to the average change value of previous studies 20.87 (Cho et al., 2004; Lee et al., 2013; Kim et al., 2014). The standard deviation $\left(\delta_{d}\right)$ of the amount of change was assumed to be 21.5, which is a common standard deviation value considering the number of samples according to previous study results (Cho et al., 2004; Lee et al., 2013; Kim et al., 2014). With a statistical power of 80% and a significance level (α) at 5%, 24 patients were required for each study arm. Assuming 20% of patients would drop out or be lost to follow-up, this study would recruit 30 participants to each group, totaling 60 participants.

### Statistical Analysis

The analysis was conducted on the intention-to-treat (ITT) population, which included all participants who completed the baseline assessment and received at least one dose of the study drug in the treatment phase but excluded participants who did not receive any study medications for various reasons. A safety analysis was performed on all participants who received at least one dose of the trial medication. The continuous variables, including the primary endpoints, were compared using an independent *t*-test. Categorical variables such as demographic characteristics were compared using Pearson’s chi-square test or Fisher’s exact test. The changes in variables over time within each group were compared using a paired *t*-test. Statistical significance was indicated by a *p*-value less than 0.05.

## Results

### Study Population

This study was conducted from June 2018 to November 2019. Thirty-six consecutive participants who fulfilled the inclusion criteria were initially enrolled; however, five participants withdrew consent due to personal reasons before starting the first drug administration. In total, 31 participants (11 male and 20 female) were included in the ITT analysis, of which 15 were assigned to receive BST and 16 to receive a placebo. The baseline demographics and clinical characteristics were similar between the two trial groups (Table 3). All participants included in the ITT analysis completed a 4 weeks clinical trial with greater than 75% adherence.

**TABLE 3:** Baseline characteristics of participants.

Measurements (Unit)	*Banha-Sasim-Tang* (*n* = 15)	Placebo (*n* = 16)	*p*-value
Age (yr)	47.07 ± 12.24	50.0 6 ± 10.82	0.61^a^
Male/Female	4/11	7/9	0.34^b^
Body weight (kg)	57.99 ± 10.67	62.36 ± 13.04	0.27^a^
Height (cm)	162.16 ± 7.48	163.53 ± 10.35	0.26^a^
BMI (kg/m^2^)	21.97 ± 3.13	22.84 ± 2.44	0.34^a^
NDI-K	79.60 ± 30.27	68.25 ± 27.32	0.52^a^
VAS (cm)	7.57 ± 1.03	6.64 ± 1.63	0.09^a^
Total ghrelin (pmol/L)	620.61 ± 429.04	515.37 ± 488.65	0.53^a^
Acyl-ghrelin (pmol/L)	166.09 ± 198.34	235.89 ± 326.30	0.48^a^
Des-acyl-ghrelin (pmol/L)	454.54 ± 348.43	279.48 ± 292.73	0.14^a^

BMI; body mass index. Scores are marked in (means ± SD).

^a^ Independent *t*-test

^b^ Pearson’s chi-square test

### Changes in Endpoints

At baseline, the mean parameters of FD symptoms were uniformly distributed in both the BST and placebo groups: NDI-K (79.60 ± 30.28 *vs* 68.25 ± 27.32), VAS (7.57 ± 1.03 *vs* 6.64 ± 1.63 cm), and total ghrelin (620.61 ± 429.04 *vs* 515.37 ± 488.65 pmol/L), respectively (Table 3).

**FIGURE 3:**
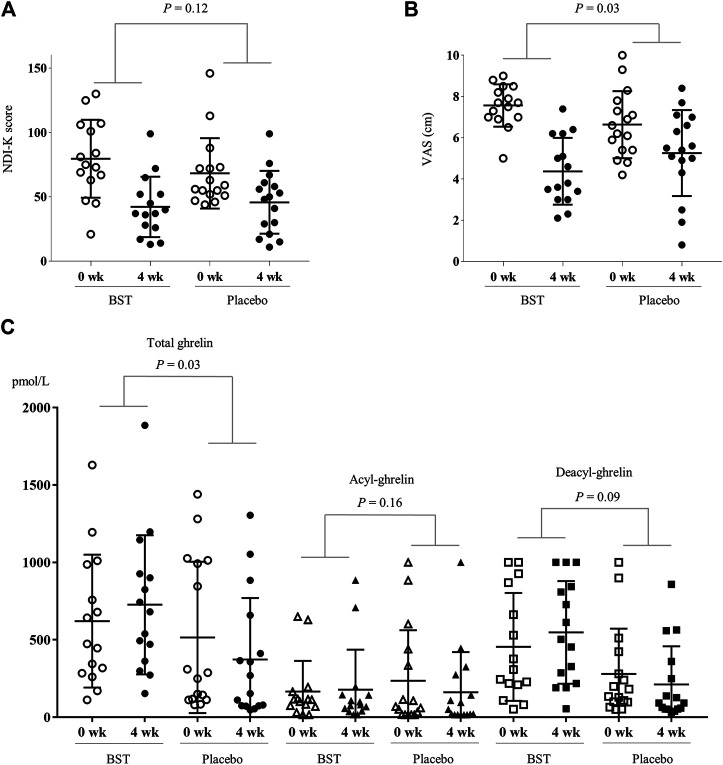
Change of measurements in BST and placebo group. The changed values were analyzed using Independent *t*-test for NDI-K score **(A)**, VAS **(B)**, and plasma ghrelin levels **(C)**.

After 4 weeks treatment, the BST group showed more improvement in NDI-K score (37.40 ± 27.40 *vs.* 22.50 ± 23.85) than the placebo group (but not statistically significant, *p* = 0.12), while the change of the VAS score was significant (3.19 ± 1.60 *vs* 1.38 ± 2.85, *p* = 0.03) (Figures 3A,B). These effects continued by 2 weeks follow-up period, especially in VAS core (3.62 ± 2.00 *vs* 1.87 ± 2.57, *p* = 0.04), (Supplementary Table S4). Also, plasma total ghrelin levels were significantly increased in the BST group compared to the placebo group (105.69 ± 287.89 pmol/L *vs* −142.31 ± 314.32 pmol/L, *p* = 0.03) (Figure 3C). BST treatment also increased both acyl and des-acyl-ghrelin levels, but they were not significant compared to the placebo group (12.29 ± 81.07 *vs* −74.56 ± 223.54 pmol/L in acyl-ghrelin, *p* = 0.16, and 93.40 ± 275.42 *vs*. −67.74 ± 226.01 pmol/L in des-acyl-ghrelin, *p* = 0.09), respectively (Figure 3C).

### Safety

One participant (6.7%) in the BST group and two participants (12.5%) in the placebo group presented some adverse events: a 65 year-old male with hematuria (urine occult blood 2+) during BST administration, while two cases of hyperbilirubinemia in a 61 year-old female (total bilirubin 1.56 mg/dl) and 38 year-old male (total bilirubin 1.54 mg/dl) in the placebo group. They all recovered over a monitoring period of 2 weeks without any treatment. No statistical correlation was observed in those adverse events with BST.

## Discussion and Conclusion

FD is a representative multifactorial and complicated pathologic disease, and various approaches have been attempted in conventional therapy, such as proton pump inhibitors (PPIs), prokinetics, and tricyclic antidepressants (TCAs) (Ahmed et al., 2018). However, these current therapeutics have many limitations including the high relapse rate up to 73% (Talley et al., 2014). As an alternative intervention, 3 clinical studies have been conducted on the effect of BST on FD to date (Yoon et al., 2003; Park et al., 2013; Zhao et al., 2013). Then, interestingly, clinical outcomes were more effective when pattern identification was adapted (Zhao et al., 2013) than when it was not adapted (Park et al., 2013). Pattern identification diagnosis and herbal prescription have received attention as personally tailored and multi-targeted medicines, especially in multifactorial diseases, in which conventional medicine already has been revealed limitations (Chiarioni et al., 2018).

The present study is the first trial to evaluate BST as an FD medication by enhancing ghrelin, especially for patients with the pathologic pattern of “liver Qi invading stomach.” According to the TKM/TCM pathology theory, excessive liver Qi, which commonly occurs under psychological stress, can invade the stomach and disrupt its homeostasis (Ling et al., 2012). Under psychological stress, the brain autonomically down-regulates gastrointestinal peristalsis and enteric endocrine system by activating the sympathetic nervous system (SNS) via releasing norepinephrine and decreasing blood flow (Kirsteen et al., 2014; Andreas and Yvette, 2010). The altered appetite, a common symptom of FD, is also caused frequently by psychological stress (Asakawa et al., 2001). Because FD is a disease of dysfunctional status without any meaningful physical defects, psychological stress is one of the most likely causes (Nan et al., 2015). In addition, the psychological stress response is also known to be associated with the secretion of ghrelin from mainly in the stomach (Andreas et al., 2011; Chuang and Zigman, 2010). Some clinical studies clearly have shown that ghrelin stimulates upper gastrointestinal motility (Tack et al., 2006) and circulating ghrelin levels could be altered by various stressors (Stengel et al., 2011). Our previous animal study found that BST modulates peristalsis via activation of the interstitial cells of Cajal and the smooth muscle cells in the stomach (Jeon et al., 2019). As we expected, BST administration notably improved the measurements of FD assessments compared with placebo, including VAS (with statistical significance) and NDI-K (Figures 3A,B). The lack of statistical significance in NDI-K might have resulted from the small number of participants. Although NDI-K just showed the tendency without statistical significance, VAS is known as a more intuitive evaluation instrument directly related to participants’ treatment satisfaction (Domenica et al., 2018).

On the other hand, BST administration significantly increased the plasma level of total ghrelin (Figure 3C). Ghrelin is a peptide hormone that is produced mainly by enteroendocrine cells of the stomach and stimulates appetite, gastrointestinal motility, and gastric acid secretion (Koji et al., 2008; Camilleri et al., 2009). Clinical studies showed lower levels of plasma total ghrelin in FD patients than in healthy volunteers (Lee et al., 2009). A significant increase in energy intake was observed with ghrelin infusion in cancer patients (Nicola et al., 2004). The intravenous administration of ghrelin promoted gastric emptying in diabetic gastroparesis patients (Murray et al., 2005). Usually, ghrelin exists in two forms, acyl-ghrelin and des-acyl-ghrelin, which are known to be active and inactive, respectively (Delporte, 2013). In our results, acyl-ghrelin and des-acyl-ghrelin were both notably increased in BST compared to placebo, but without statistical significance comparing to the placebo group. *Yukgunja-Tang* (六君子汤), another popular herbal prescription for FD patients with a *Qi* (气) deficiency pattern, also increased serum ghrelin levels as its proposed mechanism of action (Fujitsuka and Uezono, 2014). Then, *Pinellia ternate (Thunb) Makino, Panax ginseng C.A. Meyer, Zingiber officinale Roscoe, Glycyrrhiza uralensis Fisch,* and *Ziziphus jujuba Mill* overlap in preparations of both BST and *Yukgunja-Tang*. Also, *P. ternate, P. ginseng,* and *Z. officinale* directly elevated ghrelin levels in animal models (Lee and Baik, 2019). The above facts strongly suggest the modulation of ghrelin as one of the corresponding mechanisms in the improvement of FD by BST. However, the plasma ghrelin level in FD patients is still a little bit controversial. Another study reported a higher plasma ghrelin level in FD patients than in healthy volunteers (Lanzini et al., 2006).

This study has some limitations. First, we could not achieve the full recruitment of participants because of a lack of time and resources due to the investigator-initiated trial. So, the number of participants was relatively small, which might lead to the lack of statistical significance in the NDI-K score. In addition, this small number of participants gave us difficulty to analyze the gender- or age-related response rates. Second, blinding might be incomplete; likely the difference between the BST group (13/15, 86.7%) and the placebo group (6/16, 62.5%) answered the receiving BST.

Despite some limitations, this trial was the first study to claim a potential association between BST and plasma ghrelin levels in FD patients. It would be very worth finding that BST acts as a modulator of gastrointestinal ghrelin in functional dyspeptic disorders. This potential relationship should be further explored in larger studies.

## Data Availability

The original contributions presented in the study are included in the article/Supplementary Material, further inquiries can be directed to the corresponding authors.
