# Supplementary material for: Beneficial Potential of Banha-Sasim-Tang for Stress-Sensitive Functional Dyspepsia via Modulation of Ghrelin: A Randomized Controlled Trial
Source: Front Pharmacol. 2021 Apr 20;12:636752. doi: 10.3389/fphar.2021.636752 (PMC8093827; doi:10.3389/fphar.2021.636752)
Supplement: Supplementary file 1 [file table1.docx]

| **Supplementary Table 1. Questionnaire for FD pattern identification** | | |
| --- | --- | --- |
| **Please check all the following items if applicable (for participants)** | | **Agree: O, Disagree: X** |
| 1 | Epigastric bloating and stiffness |  |
| 2 | Epigastric bloating and pain |  |
| 3 | Epigastric stuffy and fullness |  |
| 4 | Subtle and persistent pain, reduced when warming up at stomach |  |
| 5 | Subtle pain and burning sensation at stomach |  |
| 6 | Full and bubbling at lower abdomen |  |
| 7 | Chest oppression |  |
| 8 | Epigastric pain stretching to flank |  |
| 9 | Dry and bitter taste in the mouth |  |
| 10 | Dry mouth and tongue |  |
| 11 | Nausea |  |
| 12 | Nausea and vomit |  |
| 13 | Loss of appetite or do not want to eat |  |
| 14 | Loss of appetite and reduced intake |  |
| 15 | Loss of taste |  |
| 16 | Feel empty stomach and no appetite even if hungry |  |
| 17 | Severe pain when hungry and reduced pain when eat food |  |
| 18 | Burping |  |
| 19 | When burp, smells like rotten eggs and regurgitates |  |
| 20 | Sighing |  |
| 21 | Pale face |  |
| 22 | Feel tired and weakness |  |
| 23 | Regurgitation |  |
| 24 | Throw up thin water |  |
| 25 | Feel heavy and languid limbs |  |
| 26 | Languid limbs with cold hand and feet |  |
| 27 | Cold hand and feet |  |
| 28 | Hot feeling of palms |  |
| 29 | Incomplete defecation or diarrhea |  |
| 30 | Watery stool and persistent diarrhea |  |
| 31 | Dry stool |  |
| 32 | Reduced urine output and yellow urine |  |
| 33 | Increased urine output and colorless urine |  |
| **Please check all the following items (for clinician)** | | **Agree: O, Disagree: X** |
| 34 | Thin and white tongue fur with pink tongue |  |
| 35 | Thick and slimy tongue fur with pink tongue |  |
| 36 | Yellow and slimy tongue fur with red tongue |  |
| 37 | Thin tongue fur with dry and red tongue |  |
| 38 | Thin and white tongue fur with pale tongue |  |
| 39 | White tongue fur and pale tongue, with teeth marked |  |
| 40 | Sunken and string like pulse |  |
| 41 | Slippery pulse |  |
| 42 | Slippery and replete pulse |  |
| 43 | Fine and rapid pulse |  |
| 44 | Rapid and string like pulse |  |
| 45 | Rapid and fine pulse |  |
