# Supplementary material for: Beneficial Potential of Banha-Sasim-Tang for Stress-Sensitive Functional Dyspepsia via Modulation of Ghrelin: A Randomized Controlled Trial
Source: Front Pharmacol. 2021 Apr 20;12:636752. doi: 10.3389/fphar.2021.636752 (PMC8093827; doi:10.3389/fphar.2021.636752)
Supplement: Supplementary file 2 [file table2.docx]

| **Supplementary Table 2. Weight score of pattern identification for FD** | | | | | |
| --- | --- | --- | --- | --- | --- |
| Liver Qi invading stomach  (肝气犯胃) | Weight score | | Dampness-heat in spleen and stomach  (脾胃湿热) | Weight score | |
| Epigastric bloating and stiffness (#1) | | 8.5 | Epigastric stuffy and fullness (#3) | | 7.0 |
| Chest oppression (#7) | | 17.5 | Dry and bitter taste in the mouth (#9) | | 12.1 |
| Epigastric pain stretching to flank (#8) | | 14.3 | Nausea and vomit (#12) | | 10.4 |
| Nausea (#11) | | 13.2 | Loss of appetite and reduced intake (#14) | | 8.0 |
| Burping (#18) | | 8.2 | Feel heavy and languid limbs (#25) | | 13.5 |
| Sighing (#20) | | 14.0 | Reduced urine output and yellow urine (#32) | | 18.4 |
| Thin and white tongue fur with pink tongue (#34) | | 9.1 | Yellow and slimy tongue fur with red tongue (#36) | | 19.6 |
| Sunken and string like pulse (#40) | | 15.3 | Slippery pulse (#41) | | 11.0 |
| Food accumulation  (飮食停滞) | Weight score | | Deficiency of stomach yin  (胃阴不足) | Weight score | |
| Epigastric bloating and pain (#2) | | 7.0 | Subtle pain and burning sensation at stomach (#5) | | 10.6 |
| Nausea and vomit (#12) | | 19.0 | Dry mouth and tongue (#10) | | 19.2 |
| Loss of appetite or do not want to eat (#13) | | 14.5 | Feel empty stomach and no appetite even if hungry (#16) | | 9.8 |
| When burp, smells like rotten eggs and regurgitates (#19) | | 23.3 | Hot feeling of palms (#28) | | 12.1 |
| Incomplete defecation or diarrhea (#29) | | 9.6 | Dry stool (#31) | | 23.1 |
| Thick and slimy tongue fur with pink tongue (#35) | | 13.4 | Thin tongue fur with dry and red tongue (#37) | | 12.5 |
| Slippery and replete pulse (#42) | | 13.3 | Fine and rapid pulse (#43) | | 12.7 |
| Cold-heat complex  (寒热错杂) | Weight score | | Spleen-stomach deficiency cold  (脾胃虚寒) | Weight score | |
| Epigastric stuffy and fullness (#3) | | 5.0 | Subtle and persistent pain, reduced when warming up at stomach (#5) | | 18.2 |
| Full and bubbling at lower abdomen (#6) | | 11.2 | Severe pain when hungry and reduced pain when eat food (#17) | | 4.6 |
| Dry and bitter taste in the mouth (#9) | | 8.2 | Pale face (#21) | | 13.5 |
| Loss of taste (#15) | | 14.7 | Feel tired and weakness (#22) | | 9.1 |
| Burping (#18) | | 9.8 | Throw up thin water (#24) | | 5.8 |
| Regurgitation (#23) | | 10.1 | Languid limbs with cold hand and feet (#26) | | 12.8 |
| Cold hand and feet (#27) | | 7.9 | Increased urine output and colorless urine (#33) | | 12.2 |
| Watery stool and persistent diarrhea (#30) | | 12.6 | White tongue fur and pale tongue, with teeth marked (#39) | | 15.5 |
| Thin and white tongue fur with pale tongue (#38) | | 9.9 | Rapid and fine pulse (#45) | | 8.3 |
| Rapid and string like pulse (#44) | | 10.6 |  | |  |
| Corresponding number that participants have agreed in the Supplementary Table 1 are classified into each pattern. Within each pattern, questions are weighted, and participants are assigned to the pattern with the highest sum of weight score | | | | | |
