# Supplementary material for: Beneficial Potential of Banha-Sasim-Tang for Stress-Sensitive Functional Dyspepsia via Modulation of Ghrelin: A Randomized Controlled Trial
Source: Front Pharmacol. 2021 Apr 20;12:636752. doi: 10.3389/fphar.2021.636752 (PMC8093827; doi:10.3389/fphar.2021.636752)
Supplement: Supplementary file 3 [file table3.docx]

| **Supplementary Table 3. NDI-K and short form of NDI-K** | | | | | | |
| --- | --- | --- | --- | --- | --- | --- |
| **NDI-K: Please mark ‘○’ on the appropriate areas if you have experienced any of the following symptoms in the past two weeks.** | | | | | | |
|  | **Frequency**  (how often you have had symptoms**)** | | **Intensity**  (how much the symptoms) | | **Bothersomeness**  (how uncomfortable with the symptom**s)** | |
| Epigastric pain | 0. Not at all  1. For 1-4 days  2. For 5-8 days  3. For 9-12 days  4. Almost every day | | 0. Not at all  1. Very weak  2. Weak  3. A bit severe  4. Severe  5. Very severe | | 0. Very weak  1. Weak  2. A bit severe  3. Severe  4. Very severe | |
| Epigastric pressure |  |  |  |  |  |  |
| Epigastric discomfort |  |  |  |  |  |  |
| Epigastric distress |  |  |  |  |  |  |
| Epigastric spasm |  |  |  |  |  |  |
| Epigastric burn |  |  |  |  |  |  |
| Heartburn |  |  |  |  |  |  |
| Regurgitation |  |  |  |  |  |  |
| Chest pain |  |  |  |  |  |  |
| Bloating |  |  |  |  |  |  |
| Nausea |  |  |  |  |  |  |
| Vomit |  |  |  |  |  |  |
| Dyspnea |  |  |  |  |  |  |
| Early satiety |  |  |  |  |  |  |
| Postprandial fullness |  |  |  |  |  |  |
| **Short form of NDI-K: Please mark ‘○’ on the appropriate areas your intensity of the following symptoms in the past two weeks.** | | | | | | |
|  | No symptom (0) | Mild (1) | | Moderate (2) | | Severe (3) |
| Epigastric pain |  |  | |  | |  |
| Epigastric pressure |  |  | |  | |  |
| Epigastric discomfort |  |  | |  | |  |
| Epigastric distress |  |  | |  | |  |
| Heartburn |  |  | |  | |  |
| Nausea |  |  | |  | |  |
| Early satiety |  |  | |  | |  |
| Postprandial fullness |  |  | |  | |  |
| The above is NDI-K, as the primary endpoint of this trial. The below is short form of NDI-K, which was used to determine if participants met the inclusion criteria when screening. | | | | | | |
