# Supplementary material for: Beneficial Potential of Banha-Sasim-Tang for Stress-Sensitive Functional Dyspepsia via Modulation of Ghrelin: A Randomized Controlled Trial
Source: Front Pharmacol. 2021 Apr 20;12:636752. doi: 10.3389/fphar.2021.636752 (PMC8093827; doi:10.3389/fphar.2021.636752)
Supplement: Supplementary file 4 [file table4.docx]

| **Supplementary Table 4. The changed values in main outcomes** | | | | | | |
| --- | --- | --- | --- | --- | --- | --- |
| Change | 4-week treatment  (pre – post) | | | 2-week follow up  (pre – f/u) | | |
|  | BST | Placebo | P-value | BST | Placebo | P-value |
| NDI-K^†^ | 20.20 ± 37.66 | 20.94 ± 20.63 | 0.12 | 42.87 ± 26.74 | 31.13 ± 28.25 | 0.68 |
| VAS^†^ | 3.20 ± 1.60 | 1.38 ± 2.85 | 0.03 | 3.62 ± 2.00 | 1.87 ± 2.57 | 0.04 |
| Scores are marked in (means ± SD). ^†^Independent t-test | | | | | | |
